# Supplementary material for: Broadening the Veterinary Consultation: Dog Owners Want to Talk about More than Physical Health
Source: Animals (Basel). 2023 Jan 24;13(3):392. doi: 10.3390/ani13030392 (PMC9913647; doi:10.3390/ani13030392)
Supplement: Supplementary file 1 [file animals-13-00392-s001.zip › animals-2054554-supplementary.pdf]

Dog owner survey: conversations at the vets

Page 1: Welcome!

Thank you for your interest in our study. Researchers at the University of Bristol Veterinary School are gathering information about the topics of conversation that dog owners have with their vets or vet nurses.

Please note: *to be eligible to complete this survey, you must be aged 18 years or over, live in the UK and currently own at least one dog.*

This survey should take you approximately 5 minutes to complete.

Data Protection and Consent

Your participation in the study will be completely confidential to all except the researchers involved. All data collected from this study will be anonymised after collection, and no participants will be identifiable in resulting reports or publications.

The data will be stored both in hard copy and electronic format and securely held at School of Veterinary Science, University of Bristol in accordance with the Data Protection Act 2018. The data will be retained for 10 years, following which it will be disposed of securely.

This study has been approved by the Bristol Veterinary School Faculty Ethics and Welfare Committee (Ref: 62063 and 63663).

1. Consent ✱ *Required*

- ○ I give consent for my data to be used as described above

2. Age ✱ *Required*

- ○ I confirm that I am 18 years of age or over

To continue, please click 'next'.

Page 2: About you and your dog(s)

In this section, we ask for a few details about you and your dog(s).

3. Please select your age category

- 18-24
- 25-34
- 35-44
- 45-54
- 55-64
- 65+

4. What is your gender?

- Male
- Female
- Other
- Prefer not to say

5. Do you work in any of the following professions relating to animal health, care, training, behaviour, or conduct research relating to dogs? If you work in more than one area, please select the job that you spend most time doing. If you do not work in any of these fields, please choose 'none of the above'.

- Vet or vet nurse
- Animal behaviourist
- Dog trainer
- Dog walker
- Animal carer
- Researcher of animal behaviour
- Other animal related profession
- None of the above

6. How many dogs do you currently own or have primary responsibility of care for?

- 1
- 2
- 3

- 4
- 5
- 6+

7. Before the dog(s) you currently own, have you previously owned or had primary responsibility for a dog?

- No, this is my first dog (or dogs)
- No, this is my first dog (or dogs) but I grew up with a dog (or dogs) in my family
- Yes, I have previously owned or co-owned a single dog
- Yes, I have previously owned or co-owned more than one dog

8. How long have you had your current dog(s)? (If you have more than one dog, please base your answer on the dog that you have had for the longest)

- 0-6 months
- 6-12 months
- 1-2 years
- 3-5 years
- 6-8 years
- 9-12 years
- 13+ years

Page 3: Experiences during visits to the vets

In this section, we will ask you about certain aspects of visits to your vets with your dog(s), which can be visits to see either a vet or a vet nurse.

9. On average, how regularly do you visit the vets with your dog(s) for an appointment with either a vet or vet nurse? **(If this year has been different because of the pandemic, please answer based on previous times).**

- Less than once a year
- Once a year
- Twice a year
- 3 - 4 times a year
- 5 or more times a year

10. Has your vet or vet nurse ever discussed the following topics about your current dog(s) with you?

	Yes	No	Unsure
Puppy socialisation or training			
Creating a safe and comfortable environment, e.g., sleeping area, travel, hazards (poisonous foods/plants)			
Your dog's body weight			
Your dog's diet/nutrition			
Neutering			
Behaviour problems			
Fear related issues			
Your dog's happiness			
Your dog's general welfare			
Your dog's quality of life			
A specific health problem			
Management of pain			
Socialising your dog with other dogs and/or people			
Mental stimulation			
Preventative medicine (e.g., worms, fleas, vaccinations)			
Senior health			
Euthanasia (end of life)			

11. How comfortable do you think you would feel discussing each of the following about your dog(s) with your vet or vet nurse?

	Very comfortable	Quite comfortable	Neither comfortable or uncomfortable	Quite uncomfortable	Very uncomfortable
Puppy socialisation or training	○	○	○	○	○
Creating a safe and comfortable environment, e.g., sleeping area, travel, hazards (poisonous foods/plants)	○	○	○	○	○
Your dog's body weight	○	○	○	○	○
Your dog's diet/nutrition	○	○	○	○	○
Neutering	○	○	○	○	○
Behaviour problems	○	○	○	○	○
Fear related issues	○	○	○	○	○
Your dog's happiness	○	○	○	○	○
Your dog's general welfare	○	○	○	○	○
Your dog's quality of life	○	○	○	○	○
A specific health problem	○	○	○	○	○
Management of pain	○	○	○	○	○
Socialising your dog with other dogs and/or people	○	○	○	○	○
Mental stimulation	○	○	○	○	○
Preventative medicine (e.g., worms, fleas, vaccination)	○	○	○	○	○
Senior health	○	○	○	○	○
Euthanasia (end of life)	○	○	○	○	○

12. When do you think these topics should be addressed by a vet or a vet nurse, if at all?

	Should be routinely discussed in every consult	Should be discussed only if owner requests	Should be discussed if vet or vet nurse thinks it's relevant	Not appropriate for vet or vet nurse to discuss	Unsure	Other	If you selected Other, please specify:
Puppy socialisation or training	○	○	○	○	○	○	

Creating a safe and comfortable environment, e.g., sleeping area, travel, hazards (poisonous foods/plants)							
Your dog's body weight							
Your dog's diet/nutrition							
Neutering							
Behaviour problems							
Fear related issues							
Your dog's happiness							
Your dog's general welfare							
Your dog's quality of life							
A specific health problem							
Management of pain							
Socialising your dog with other dogs and/or people							
Mental stimulation							
Preventative medicine (e.g., worms, fleas, vaccinations)							
Senior health							
Euthanasia (end of life)							

13. How do you think these topics should be addressed by a vet or a vet nurse, if at all?

	Within a routine 10-15 minute consultation	Within a separate, specific 10-15 minute consultation	Not appropriate for vet or vet nurse to discuss	Unsure	Other	If you selected Other, please specify:
Puppy socialisation or training	○	○	○	○	○	
Creating a safe and comfortable environment, e.g., sleeping area, travel, hazards (poisonous foods/plants)	○	○	○	○	○	
Your dog's body weight	○	○	○	○	○	
Your dog's diet/nutrition	○	○	○	○	○	
Neutering	○	○	○	○	○	
Behaviour problems	○	○	○	○	○	
Fear related issues	○	○	○	○	○	
Your dog's happiness	○	○	○	○	○	
Your dog's general welfare	○	○	○	○	○	
Your dog's quality of life	○	○	○	○	○	
A specific health problem	○	○	○	○	○	
Management of pain	○	○	○	○	○	
Socialising your dog with other dogs and/or people	○	○	○	○	○	
Mental stimulation	○	○	○	○	○	

Preventative medicine (e.g., worms, fleas, vaccinations)	○	○	○	○	○	
Senior health	○	○	○	○	○	
Euthanasia (end of life)	○	○	○	○	○	

14. If you could only choose 3 aspects about your dog's **current life** to discuss with your vet or vet nurse, which would they be?

Please select between 1 and 3 answers.

- ☐ Puppy socialisation or training
- ☐ Creating a safe and comfortable environment, e.g., sleeping area, travel, hazards (poisonous foods/plants)
- ☐ Your dog's body weight
- ☐ Your dog's diet/nutrition
- ☐ Neutering
- ☐ Behaviour problems
- ☐ Fear related issues
- ☐ Your dog's happiness
- ☐ Your dog's general welfare
- ☐ Your dog's quality of life
- ☐ A specific health problem
- ☐ Management of pain
- ☐ Socialising your dog with other dogs and/or people
- ☐ Mental stimulation
- ☐ Preventative medicine (e.g., worms, fleas, vaccinations)
- ☐ Senior health
- ☐ Euthanasia (end of life)
- ☐ None of the above

15. If there is a topic that does not appear on this list, which you would like to discuss with your vet or vet nurse, please provide details:

16. Has your vet or vet nurse ever used a paper or computerised questionnaire to ask about your dog’s health or wellbeing, or asked you to complete one?

- Yes
- No
- Unsure

16.a. If you answered 'yes', please state the type/purpose of the questionnaire. For example: 'improving care', 'treatment evaluation', 'checking overall wellbeing', 'decision-making, e.g. end of life', or 'I don't know' if you are unsure

16.b. If you answered 'yes', how useful did you find this?

17. Have you ever used an app to complete questions about your dog's health or wellbeing, e.g. PetDialog app?

- Yes
- No
- Unsure

17.a. If you answered 'yes', please state the type/purpose of the questionnaire. For example: 'improving care', 'treatment evaluation', 'checking overall wellbeing', 'decision-making, e.g. end of life', or 'I don't know' if you are unsure

17.b. If you answered 'yes', how useful did you find this?

18. If widely available, would you wish to access tools designed to assess different aspects of your dog's behaviour, health, or wellbeing that you could complete in your own time?

- Yes
- No
- Unsure

19. What does the term 'quality of life' mean to you, in relation to your dog(s)?

Page 4: Further research

20. We are interested in speaking to some owners over the telephone to ask a few further questions on this research topic. Would you like to be considered for this part of our study?

- ○ Yes please
- ○ No thank you

20.a. If you answered yes, please provide your email and telephone contact details below. *Any details provided will be kept strictly confidential and will not be shared outside this study.*

Page 5: Thank you for your time

Thank you for completing this survey.

Your responses will help us better understand dog owner opinions and experiences of vet visits.

If you would like to contact us with any questions or query relating to this survey, please email:
helena.hale@bristol.ac.uk

Key for selection options

16.b - If you answered 'yes', how useful did you find this?
- Extremely useful
- Quite useful
- Unsure
- Quite unuseful
- Extremely unuseful

17.b - If you answered 'yes', how useful did you find this?
- Extremely useful
- Quite useful
- Unsure
- Quite unuseful
- Extremely unuseful
